# Dose-dependent white matter changes associated with repetitive head impacts in former American football players

**DOI:** 10.1093/braincomms/fcag195

**Published:** 2026-05-31

**Authors:** Hector Arciniega, Alana Wickham, Brian Szekely, Nicholas Kim, Kang I Cho, Holly Carrington, Evdokiya E Knyazhanskaya, Omar John, Leonard B Jung, Katherine Breedlove, Anya S Mirmajlesi, Jared Stearns, Richard Jarrett Rushmore, Daniel H Daneshvar, Tim L T Wiegand, Tashrif Billah, Ofer Pasternak, Suheyla Cetin-Karayumak, Yogesh Rathi, Michael J Coleman, Charles H Adler, Charles Bernick, Laura J Balcer, Brian S Im, Shae Datta, Michael L Alosco, Inga K Koerte, Alexander P Lin, Jeffrey L Cummings, Eric M Reiman, Robert A Stern, Martha E Shenton, Sylvain Bouix, DIAGNOSE CTE Research Project, Eric Reiman, Yi Su, Kewei Chen, Hillary Protas, Connie Boker, Michael L Alosco, Rhoda Au, Robert C Cantu, Lindsay Farrer, Robert Helm, Douglas I Katz, Neil Kowall, Jesse Mez, Gustavo Mercier, James Otis, Robert A Stern, Jason Weller, Irene Simkin, Alondra Andino, Shannon Conneely, Courtney Diamond, Tessa Fagle, Olivia Haller, Tennyson Hunt, Nicole Gullotti, Megan Mariani, Brian Mayville, Kathleen McLaughlin, Mary Nanna, Taylor Platt, Surya Pulukuri, Fiona Rice, Madison Sestak, Michael McClean, Yorghos Tripodis, Douglas Annis, Christine Chaisson, Diane B Dixon, Carolyn Finney, Kerrin Gallagher, Kaitlin Hartlage, Jun Lu, Brett Martin, Emmanuel Ojo, Joseph N Palmisano, Brittany Pine, Janani Ramachandran, Sylvain Bouix, Jennifer Fitzsimmons, Alexander P Lin, Inga K Koerte, Ofer Pasternak, Martha E Shenton, Hector Arcinieago, Tashrif Billah, Elena Bonke, Katherine Breedlove, Eduardo Coello, Michael J Coleman, Leonhard Jung, Huijun Liao, Maria Loy, Elizabeth Rizzoni, Vivian Schultz, Annelise Silva, Brynn Vessey, Tim L T Wiegand, Sarah Banks, Charles Bernick, Jason Miller, Aaron Ritter, Marwan Sabbagh, Raelynn de la Cruz, Jan Durant, Morgan Golceker, Nicolette Harmon, Kaeson Kaylegian, Rachelle Long, Christin Nance, Priscilla Sandoval, Robert W Turner, Kenneth L Marek, Andrew Serrano, Charles H Adler, David W Dodick, Yonas Geda, Jennifer V Wethe, Bryce Falk, Amy Duffy, Marci Howard, Michelle Montague, Thomas Osgood, Debra Babcock, Patrick Bellgowan, Laura Balcer, William Barr, Judith Goldberg, Thomas Wisniewski, Ivan Kirov, Yvonne Lui, Charles Marmar, Lisena Hasanaj, Liliana Serrano, Alhassan Al-Kharafi, Allan George, Sammie Martin, Edward Riley, William Runge, Jeffrey L Cummings, Elaine R Peskind, Elizabeth Colasurdo, Daniel S Marcus, Jenny Gurney, Richard Greenwald, Keith A Johnson

**Affiliations:** Department of Rehabilitation Medicine, NYU Grossman School of Medicine, NewYork, NY 10016, USA; NYU Langone Concussion Center, NYU Langone Health, NewYork, NY 10016, USA; Department of Rehabilitation Medicine, NYU Grossman School of Medicine, NewYork, NY 10016, USA; NYU Langone Concussion Center, NYU Langone Health, NewYork, NY 10016, USA; Psychiatry Neuroimaging Laboratory, Brigham and Women’s Hospital, Harvard Medical School, Boston, MA 02145, USA; Department of Rehabilitation Medicine, NYU Grossman School of Medicine, NewYork, NY 10016, USA; NYU Langone Concussion Center, NYU Langone Health, NewYork, NY 10016, USA; Psychiatry Neuroimaging Laboratory, Brigham and Women’s Hospital, Harvard Medical School, Boston, MA 02145, USA; Psychiatry Neuroimaging Laboratory, Brigham and Women’s Hospital, Harvard Medical School, Boston, MA 02145, USA; Psychiatry Neuroimaging Laboratory, Brigham and Women’s Hospital, Harvard Medical School, Boston, MA 02145, USA; Psychiatry Neuroimaging Laboratory, Brigham and Women’s Hospital, Harvard Medical School, Boston, MA 02145, USA; Department of Rehabilitation Medicine, NYU Grossman School of Medicine, NewYork, NY 10016, USA; NYU Langone Concussion Center, NYU Langone Health, NewYork, NY 10016, USA; Psychiatry Neuroimaging Laboratory, Brigham and Women’s Hospital, Harvard Medical School, Boston, MA 02145, USA; Psychiatry Neuroimaging Laboratory, Brigham and Women’s Hospital, Harvard Medical School, Boston, MA 02145, USA; Department of Neurosurgery, University Hospital, Ludwig-Maximilians-Universität, Munich 80336, Germany; cBRAIN, Department of Child and Adolescent Psychiatry, Psychosomatics, and Psychotherapy, University Hospital, Ludwig-Maximilians-Universität, Munich 80336, Germany; Center for Clinical Spectroscopy, Department of Radiology, Brigham and Women’s Hospital, Harvard Medical School, Boston, MA 02115, USA; Department of Rehabilitation Medicine, NYU Grossman School of Medicine, NewYork, NY 10016, USA; NYU Langone Concussion Center, NYU Langone Health, NewYork, NY 10016, USA; Department of Rehabilitation Medicine, NYU Grossman School of Medicine, NewYork, NY 10016, USA; NYU Langone Concussion Center, NYU Langone Health, NewYork, NY 10016, USA; Psychiatry Neuroimaging Laboratory, Brigham and Women’s Hospital, Harvard Medical School, Boston, MA 02145, USA; Department of Anatomy and Neurobiology, Chobanian and Avedisian School of Medicine, Boston University, Boston, MA 02118, USA; Center for Morphometric Analysis, Department of Neurology and Psychiatry, Massachusetts General Hospital, Charlestown, MA 02145, USA; Department of Physical Medicine and Rehabilitation, Harvard Medical School, Boston, MA 02115, USA; Department of Physical Medicine and Rehabilitation, Massachusetts General Hospital, Boston, MA 02114, USA; Department of Physical Medicine and Rehabilitation, Spaulding Rehabilitation Hospital, Boston, MA 02129, USA; Psychiatry Neuroimaging Laboratory, Brigham and Women’s Hospital, Harvard Medical School, Boston, MA 02145, USA; cBRAIN, Department of Child and Adolescent Psychiatry, Psychosomatics, and Psychotherapy, University Hospital, Ludwig-Maximilians-Universität, Munich 80336, Germany; Computational Neurology, Department of Neurology, Charité-Universitätsmedizin Berlin, Berlin 10117, Germany; Computational Neurology, Berlin Institute of Health, Berlin 10117, Germany; Psychiatry Neuroimaging Laboratory, Brigham and Women’s Hospital, Harvard Medical School, Boston, MA 02145, USA; Psychiatry Neuroimaging Laboratory, Brigham and Women’s Hospital, Harvard Medical School, Boston, MA 02145, USA; Department of Radiology, Brigham and Women’s Hospital, Harvard Medical School, Boston, MA 02115, USA; Department of Psychiatry, Massachusetts General Hospital, Boston, MA 02114, USA; Psychiatry Neuroimaging Laboratory, Brigham and Women’s Hospital, Harvard Medical School, Boston, MA 02145, USA; Department of Radiology, Brigham and Women’s Hospital, Harvard Medical School, Boston, MA 02115, USA; Psychiatry Neuroimaging Laboratory, Brigham and Women’s Hospital, Harvard Medical School, Boston, MA 02145, USA; Department of Radiology, Brigham and Women’s Hospital, Harvard Medical School, Boston, MA 02115, USA; Department of Psychiatry, Massachusetts General Hospital, Boston, MA 02114, USA; Psychiatry Neuroimaging Laboratory, Brigham and Women’s Hospital, Harvard Medical School, Boston, MA 02145, USA; Department of Neurology, Mayo Clinic College of Medicine, Mayo Clinic Arizona, Scottsdale, AZ 85259, USA; Cleveland Clinic Lou Ruvo Center for Brain Health, Las Vegas, NV 89106, USA; Department of Neurology, University of Washington, Seattle, WA 98195, USA; Department of Neurology, NYU Grossman School of Medicine, NewYork, NY 10016, USA; Department of Population Health, NYU Grossman School of Medicine, NewYork, NY 10017, USA; Department of Ophthalmology, NYU Grossman School of Medicine, NewYork, NY 10017, USA; Department of Rehabilitation Medicine, NYU Grossman School of Medicine, NewYork, NY 10016, USA; NYU Langone Concussion Center, NYU Langone Health, NewYork, NY 10016, USA; NYU Langone Concussion Center, NYU Langone Health, NewYork, NY 10016, USA; Department of Neurology, NYU Grossman School of Medicine, NewYork, NY 10016, USA; Department of Neurology, Boston University Alzheimer’s Disease Research Center and CTE Center, Boston University Chobanian & Avedisian School of Medicine, Boston, MA 02118, USA; Psychiatry Neuroimaging Laboratory, Brigham and Women’s Hospital, Harvard Medical School, Boston, MA 02145, USA; cBRAIN, Department of Child and Adolescent Psychiatry, Psychosomatics, and Psychotherapy, University Hospital, Ludwig-Maximilians-Universität, Munich 80336, Germany; Department of Psychiatry, Massachusetts General Hospital, Boston, MA 02114, USA; Graduate School of Systemic Neurosciences, Ludwig-Maximilians-Universität, Munich, Bavaria 82152, Germany; Center for Clinical Spectroscopy, Department of Radiology, Brigham and Women’s Hospital, Harvard Medical School, Boston, MA 02115, USA; Department of Radiology, Brigham and Women’s Hospital, Harvard Medical School, Boston, MA 02115, USA; Chambers-Grundy Center for Transformative Neuroscience, Department of Brain Health, Kirk Kerkorian School of Medicine, University of Nevada, Las Vegas, Las Vegas, NV 89154, USA; Banner Alzheimer’s Institute, Arizona State University, and Arizona Alzheimer’s Consortium, Phoenix, AZ 85006, USA; Department of Psychiatry, University of Arizona, Phoenix, AZ 85004, USA; Department of Psychiatry, Arizona State University, Phoenix, AZ 85008, USA; Neurogenomics Division, Translational Genomics Research Institute and Alzheimer’s Consortium, Phoenix, AZ 85004, USA; Department of Anatomy and Neurobiology, Chobanian and Avedisian School of Medicine, Boston University, Boston, MA 02118, USA; Department of Neurology, Boston University Alzheimer’s Disease Research Center and CTE Center, Boston University Chobanian & Avedisian School of Medicine, Boston, MA 02118, USA; Department of Neurosurgery, Boston University Chobanian & Avedisian School of Medicine, Boston, MA 02118, USA; Psychiatry Neuroimaging Laboratory, Brigham and Women’s Hospital, Harvard Medical School, Boston, MA 02145, USA; Department of Physical Medicine and Rehabilitation, Harvard Medical School, Boston, MA 02115, USA; Department of Physical Medicine and Rehabilitation, Massachusetts General Hospital, Boston, MA 02114, USA; Department of Software Engineering and Information Technology, École de technologie supérieure, Université du Québec, Montréal, QC, Canada H3C 1K3

**Keywords:** neuroimaging, sports-related head injury, repetitive head impact, American football, diffusion tensor imaging

## Abstract

Repetitive head impacts sustained during American football have been associated with neuropathological changes such as white matter shear injuries. However, the impact of specific factors, such as age of first exposure and cumulative head impact burden, on white matter integrity remains unclear. This study investigated *in vivo* white matter microstructural changes using diffusion tensor imaging and tract-based spatial statistics in 165 male former American football players (mean age 57.3 years, range 45–74) and 52 unexposed asymptomatic male controls (mean age 59.4 years, range 45–74) in the DIAGNOSE CTE Research Project. Compared to controls, former football players exhibited significantly higher fractional anisotropy (FA) in 1.97% of the white matter skeleton (1552 voxels; Cohen’s *d* = 0.587) and higher tissue-corrected FA (FAt) in 1.48% of the white matter skeleton (1004 voxels; Cohen’s *d* = 0.616). No significant differences were observed for mean diffusivity, axial diffusivity, radial diffusivity, or free water between football players and controls. Among football players, there were no significant differences in the white matter microstructure between players diagnosed with traumatic encephalopathy syndrome and those without the diagnosis. Lower FA was significantly associated with older age (*P* < 0.00001) and an earlier age of first exposure to tackle football (*P* < 0.01), while lower FAt was associated with greater cumulative head impact burden, specifically higher linear acceleration (*P* < 0.04) and rotational force (*P* < 0.02). This study highlights the influential role of exposure factors on white matter microstructure in former American football players, as well as the utility of diffusion tensor imaging to aid in characterizing the long-term effects of repetitive head impacts in contact sport athletes.

## Teaser

Arciniega *et al*. report that former American football players show modest, spatially restricted alterations in white matter microstructure compared with unexposed controls using diffusion MRI. These differences relate to age and exposure history, but do not distinguish players with or without traumatic encephalopathy syndrome.

## Graphical Abstract

**Graphical Abstract:**
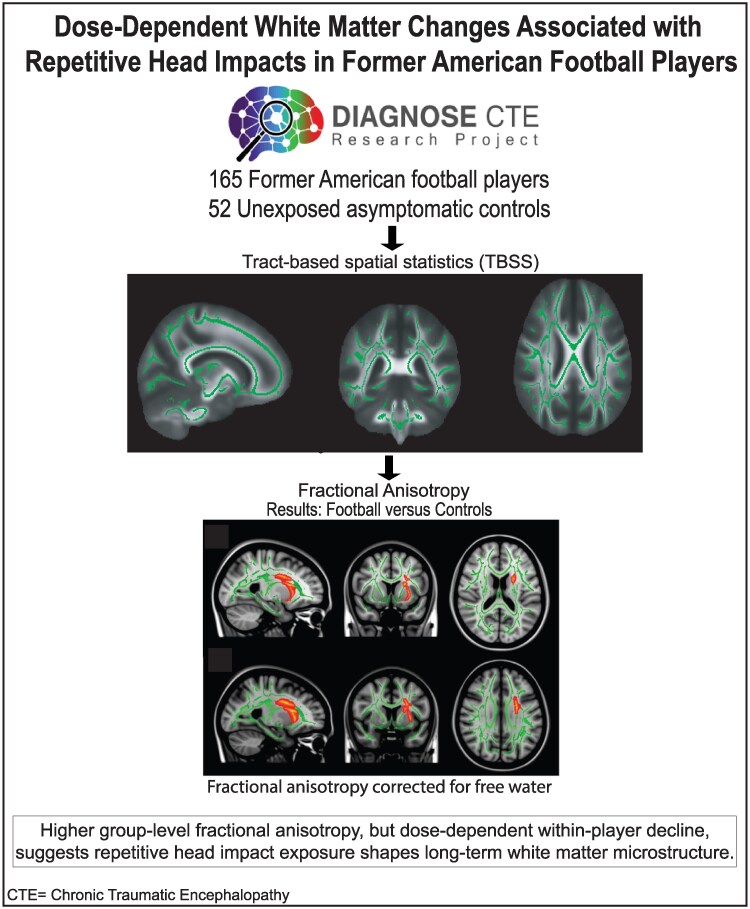

## Introduction

Division I collegiate American football players can experience up to 1444 head impacts in a single season, averaging 6.3 impacts per practice and 14.3 per game.^1^ These forms of repetitive head impacts (RHIs), defined as sub-concussive blows to the head that do not meet the criteria for concussion or mild traumatic brain injury (TBI), have been associated with long-term cognitive decline, mood and behavioural disturbances, as well as increased risk for neurodegenerative diseases such as chronic traumatic encephalopathy (CTE).^2-17^ Increasing evidence suggests that the timing, frequency and severity of RHI exposure are critical factors that shape both clinical outcomes and underlying neuroanatomical change.^4,8,18-21^ For example, an earlier age of exposure to tackle football has been linked to more significant executive dysfunction, memory deficits and reduced thalamic volume later in life.^8,22-24^ Furthermore, the cumulative burden of RHI has been shown to predict later-life cognitive impairment, neuropsychiatric symptoms and an increased risk for developing CTE.^8,25,26^ These clinical manifestations may be driven by underlying neuropathological changes that may include white matter shear injury, cortical thinning and/or cerebral atrophy.^27,28^

One approach to investigating white matter microstructure is to use diffusion tensor imaging (DTI), a technique that measures the diffusion of water molecules through brain tissue to assess microstructural integrity. DTI yields several key metrics, including fractional anisotropy (FA), mean diffusivity (MD), axial diffusivity (AD), radial diffusivity (RD) and, with further processing, FA corrected for free water (FAt).^29^ FA reflects the directionality of water diffusion, with higher values indicating more organized white matter. In contrast, FAt, derived from free-water imaging, removes extracellular free water from the tissue-specific signal, providing a more precise measure of axonal integrity.^29,30^ AD and RD provide insights into axonal and myelin health, respectively, while MD reflects the overall magnitude of water diffusion. In the context of RHI, DTI offers a sensitive tool for detecting microstructural white matter alterations that may reflect early signs of injury, including diffuse axonal damage, demyelination and neuroinflammation. Reductions in AD tend to indicate diffuse axonal injury, increased RD implies potential demyelination, and higher overall MD may suggest greater levels of inflammation. These microstructural alterations may contribute to the development of long-term neurological conditions associated with RHI, such as CTE.^31^ These insights can also help refine the clinical framework for traumatic encephalopathy syndrome (TES), a research-based diagnosis designed to characterize the constellation of symptoms observed during life in individuals with a history of RHI and suspected of having CTE.^32^ Therefore, by capturing subtle changes in diffusion metrics, DTI can help identify patterns of white matter disruption associated with cumulative exposure to head trauma, providing insight into the mechanisms of injury and potential biomarkers of disease.

Prior diffusion MRI studies of sport-related head injury demonstrate heterogeneous findings that vary by injury chronicity, cohort characteristics and analytic approach.^33,34^ In the acute and subacute period after concussion, diffusion alterations are often interpreted in the context of oedema, neuroinflammation and evolving axonal physiology and may include regionally increased or decreased FA alongside changes in diffusivity metrics.^35-37^ In contrast, studies of former athletes with long-term exposure to RHI have reported mixed patterns, including lower FA in some cohorts and elevated FA in select tracts in others, underscoring that diffusion-derived measures can reflect multiple microstructural processes rather than a single injury signature.^19,35,38-42^ Additionally, models that separate extracellular free water from tissue signal (e.g. free-water imaging) may provide complementary sensitivity to chronic microstructural alterations by reducing partial-volume effects and disentangling tissue-specific changes from extracellular contributions.^29^ Together, this literature motivates the present study’s focus on both conventional tensor metrics and tissue-corrected FA and supports the evaluation of exposure timing and cumulative impact burden as key sources of inter-individual variability.

In this study, we leveraged DTI to investigate *in vivo* alterations in white matter microstructure among former American football players with a history of RHI. Our primary objective was to determine whether former football players exhibit significant differences in whole-brain diffusion metrics compared to unexposed asymptomatic controls, highlighting potential long-term neurobiological consequences of RHI. We examined whether diffusion profiles differ between former football players diagnosed with TES and those without TES. Finally, we assessed how age, age of first exposure to tackle football and cumulative head impact burden relate to DTI-derived measures of white matter integrity. We hypothesized that RHI exposure would be associated with lower FA, FAt and AD and higher RD and MD compared to controls; that TES diagnosis would be linked to more pronounced microstructural abnormalities; and that earlier exposure, greater head impact burden and older age would predict greater white matter disruption. It was anticipated that these findings would clarify the relationship between RHI and white matter pathology and inform future strategies for early identification and prevention of long-term neurological consequences in contact sports athletes.

## Materials and methods

### Study design and participants

This study used baseline data from the Diagnostic, Imaging, And Genetics Network for the Objective Study and Evaluation of Chronic Traumatic Encephalopathy (DIAGNOSE CTE) Research Project,^43^ a multi-site study designed to identify *in vivo* markers associated with CTE and RHI exposure. Participants were enrolled at Boston University School of Medicine, NYU Langone Health, Cleveland Clinic Lou Ruvo Center for Brain Health and Mayo Clinic Arizona. Institutional review board approval was obtained at each participating centre, and all participants provided written informed consent before enrolment. The broader study design and eligibility procedures have been described previously.^43^

The parent cohort included 240 men: 180 former American football players and 60 asymptomatic unexposed control participants without a history of RHI or TBI. For the present analysis, 14 former football players and 4 controls were excluded because of poor-quality and/or incomplete structural MRI data. An additional four controls were excluded after follow-up revealed prior RHI exposure or longstanding psychiatric conditions. The final analytic sample, therefore, consisted of 165 former football players (111 former professional players and 54 former collegiate players) and 52 unexposed asymptomatic controls (see Table 1 for cohort demographics). Supplementary Fig. 1 gives details on inclusion/exclusion criteria and sample exclusion based on data quality.

**Table 1:** Cohort characteristics

	Former American football players (*n* = 165)	Unexposed asymptomatic controls (*n* = 52)	*P*-values
Primary demographics			
Age	57.3 y (8.2), [45 y–74 y]	59.4 y (8.5), [45 y–74 y]	0.12
BMI, kg/m^2^	32.6 (4.7), [22.8–47.4]	30.88 (4.7), [23.7–43.5]	0.02
Education	16.8 y (1.5), [15 y–27 y]	17 y (3.0), [13 y–30 y]	0.49
Apolipoprotein 4 carriers	45 (27.3%)	9 (17.3%)	0.34
Race			0.43
White	105 (63.6%)	32 (61.5%)	
Black/African American	57 (34.5%)	19 (36.5%)	
American Indian/Alaska	0 (0%)	0 (0%)	
Native			
Asian	0 (0%)	0 (0%)	
Native Hawaiian/Other	0 (0%)	1 (1.9%)	
Pacific Islander			
Multiple races	3 (1.9%)	0 (0%)	
Unknown	0 (0%)	0 (0%)	
Exposure to RHIs			
Number of years in football	16 y (4.4), [6 y–25 y]		
Age of first exposure	11.1 y (2.9), [4 y–18 y]		
Cumulative head impact index seasons			
Frequency	10 925 (4713), [3560–28 020]		
Linear acceleration	228 773 (73 637), [79 212–446 257]		
Rotational force	1 8403 853 (6 473 953), [6 053 874–44 072 194]		
Traumatic encephalopathy syndrome			
Traumatic encephalopathy syndrome diagnosis (%)	105 (64%)	0 (0%)	
Sub-category: cognitive impairment (%)	96 (58%)	5 (9%)	
Sub-category: neurobehavioural dysregulation (%)	94 (57%)	1 (1.8%)	
Sub-category: cognitive impairment and neurobehavioural dysregulation (%)	62 (38%)	0 (0%)	

Overview of cohort characteristics, including demographics of 165 former American football players and 52 unexposed asymptomatic control participants. Apolipoprotein 4 carrier data were only available for 210 participants. Diagnosis of traumatic encephalopathy syndrome (cognitive impairment and neurobehavioural dysregulation) was completed on all participants. Values represent (mean, standard deviation, [range]).

y, years.

### Sample characteristics

Participant information was obtained through semi-structured interviews and online questionnaires. Variables collected included demographic characteristics, medical and psychiatric history, athletic history, military service and TBI history. Age was treated as a continuous variable, and education was quantified as total years completed. Race and ethnicity were categorized according to the Office of Management and Budget Directive 15 definitions, with participants permitted to endorse more than one category. Body mass index (BMI) was calculated from measured height and weight. Whole-blood samples were also obtained for apolipoprotein ε4 (*APOE* ε4) genotyping (see Table 1 for cohort characteristics).

### Exposure to repetitive head impact

Among former football players, exposure history was characterized using age at first exposure to tackle football and the cumulative head impact index (CHII). The CHII incorporates the number of seasons played, playing position at each level of competition, and previously established helmet accelerometry estimates for that position.^25^ Separate cumulative estimates were derived for impact frequency, linear acceleration and rotational acceleration, with higher values indicating greater lifetime exposure burden.^25^

### Evaluation of traumatic encephalopathy syndrome

All participants underwent diagnostic adjudication for TES using a multidisciplinary consensus conference based on the 2021 NINDS consensus criteria.^32^ Information reviewed by the panel included medical history; RHI exposure; participant- and informant-reported cognitive, mood and behavioural symptoms; functional status; neurological and motor findings; and standardized neuropsychological and neuropsychiatric testing results.^32^

### Image acquisition

All participants underwent MRI at one of the four study sites using the same scanner platform and acquisition protocol. Imaging was performed on 3T Siemens Magnetom Skyra systems running VE11 software and equipped with 20-channel head coils. The study’s multimodal imaging protocol included structural, diffusion and resting-state functional MRI sequences. The multi-shell diffusion MRI (dMRI) data used for our analysis was acquired using the GeneRalized Autocalibration Partial Parallel Acquisition (GRAPPA) sequence (TR = 11 000 ms, TE = 105 ms, 30 gradient directions at b = 2500/mm^2^, 30 at b = 1000 s/mm^2^, 6 at b = 500 s/mm^2^, 3 at b = 200 s/mm^2^ and 5 interleaved b0 images, 2 × 2 × 2 mm^3^ voxel size). The 30 gradient directions at b = 2500 s/mm^2^ were collected for tractography studies and were not used for tensor estimation (see below) to prevent non-Gaussian effects.

### Image analysis

All scans underwent quality control by trained raters at the Psychiatry Neuroimaging Laboratory. Raw diffusion data were visually reviewed for artefacts such as signal dropout, blur and ghosting. Motion and eddy-current-related distortions were corrected within the laboratory’s processing pipeline^44^ using FSL-based registration tools.^45^ From these transformations, an average between-volume displacement metric was derived, and diffusion gradient directions were adjusted to account for rotational components of motion correction. Diffusion-weighted masks were manually refined in 3D Slicer. Diffusion-weighted imaging masks were manually edited in 3D Slicer version 4.10.2.^46^ Data were then harmonized across the four sites using a retrospective multi-shell dMRI data harmonization algorithm.^47,48^ In this procedure, one scanner is chosen as a reference (in our case, the Brigham and Women’s Hospital site), and a voxel-level mapping between each site and the reference is estimated using a set of demographically matched healthy controls between each target site and the reference. These mappings are then used to harmonize the remaining subjects at each site, ensuring consistency across scanners.^47^

A non-linear fit estimate of diffusion tensors generated FA maps using FSL. Additionally, we conducted a free-water processing method to separate the properties of brain tissue from surrounding free water.^29^ Measures obtained from this model, and used in this study, were the free-water compartment fraction (FW) and the tissue-FA (FAt).

Tract-based spatial statistics (TBSS) were used to analyse FA, FW, FAt, MD, RD and AD measures. We processed our data with an optimized TBSS workflow developed at the Psychiatry Neuroimaging Laboratory.^49^

### Statistical analysis

To examine group-level differences in demographic variables between former American football players and unexposed asymptomatic control participants, we conducted an independent Welch’s *t*-test for continuous variables (age, BMI and education) and applied a Fisher’s exact test for categorical variables (race and APOE ε4 gene status).

We performed group comparisons for each voxel on the white matter skeleton to identify the spatial extent of group differences, using the Randomise Tool in FSL.^50^ We performed non-parametric permutation-based F tests, testing for group differences in FA, MD, AD, RD, FAt and FW separately. We used age, education, BMI, race and e4 carrier status as covariates. Threshold-free cluster enhancement^51^ was applied to control for family-wise error arising from whole-brain voxel-wise multiple comparisons. Statistical significance was defined at *P* < 0.01 after family-wise error correction. This conservative threshold was selected to reduce false-positive findings in the context of whole-brain diffusion analyses. Significant voxel-wise test maps provide clusters of voxels that demonstrate the locations of significant between-group differences, and anatomical locations were identified using the JHU-ICBM-DTI-81 Atlas.^52^

Within the former football player cohort, separate linear regression models were used to examine associations between diffusion outcomes and exposure-related variables. FA and FAt were each modelled as dependent variables in separate analyses, with age, age of first exposure to tackle football and CHII (frequency, linear acceleration and rotational force) evaluated in independent models. In total, 10 regression models were performed (two diffusion outcomes × five exposure predictors). All *P*-values were corrected for multiple comparisons using the Benjamini–Hochberg procedure, with statistical significance defined as *P* < 0.05. For these regression analyses, FA and FAt values were averaged across the entire TBSS white matter skeleton and extracted for each participant before modelling.

## Results

To examine group-level differences in demographic variables between former American football players (*n* = 165) and unexposed asymptomatic control participants (*n* = 52), we conducted two-tailed Welch’s two-sample *t*-tests for continuous variables (age, BMI and education years) and Fisher’s exact tests for categorical variables (race and APOE ε4 carrier status). Groups did not differ in age (*P* = 0.12), education (*P* = 0.49), APOE ε4 carrier status (*P* = 0.34), or race distribution (*P* = 0.43); however, former football players had significantly higher BMI (*P* = 0.02).

### Demographical characteristics

Using a Welch two-sample *t*-test, we detected differences in BMI between our former American football players and unexposed asymptomatic controls [t(85.94) = 2.45, mean difference, 1.83, 95% CI (0.35, 3.31), *P* = 0.016], signifying a higher BMI in the football player group. Using a *χ*² test, we detected no significant differences in race (*P* = 0.429) or APOE ε4 gene status (*P* = 0.342) between groups. No additional group differences were evident (see Table 1 for demographic breakdowns).

### Voxel-wise analysis

**Figure 1:**
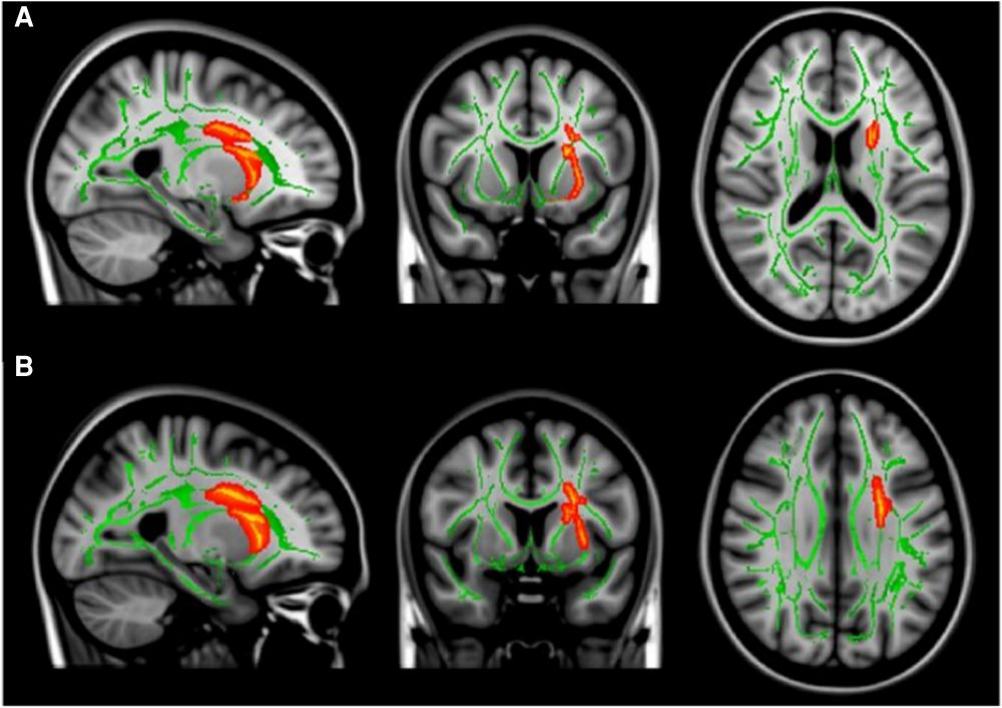
Former American football players (*n* = 165) have significantly higher FA and FAt than unexposed asymptomatic controls (*n* = 52) in the left hemisphere. (**A**) TBSS analysis of FA and (**B**) FAt. Highlighted regions represent significantly higher FA and FAt in former football players compared to controls (*P* < 0.01, family-wise error-corrected using threshold-free cluster enhancement), superimposed on a template image and the average white matter skeleton across all subjects (green). Red-yellow clusters indicate the location of significant voxel-wise group differences, with colour intensity reflecting the magnitude of the test statistic. The right side of the image corresponds to the left hemisphere of the brain. FA, fractional anisotropy; FAt, tissue-corrected fractional anisotropy; TBSS, tract-based spatial statistics.

**Figure 2:**
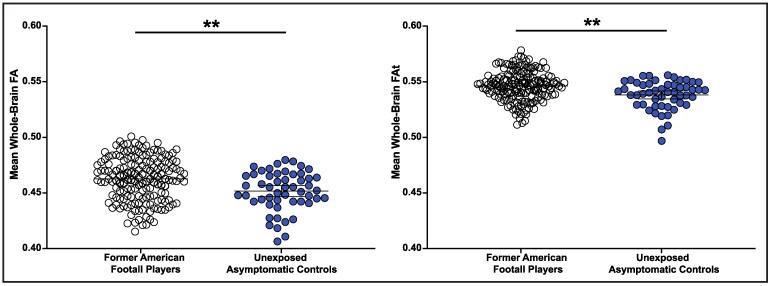
Distribution of whole-brain diffusion metrics in former American football players and unexposed asymptomatic controls. Individual-level distributions of mean whole-brain FA (left) and (right) are shown for former American football players (*n* = 165) and unexposed asymptomatic controls (*n* = 52). Each point represents an individual participant; horizontal lines indicate group means. Group differences were assessed using non-parametric permutation-based F tests controlling for age, education, BMI, race and APOE ε4 carrier status. *P* < 0.01. FA, fractional anisotropy; FAt, tissue-corrected fractional anisotropy; BMI, body mass index; APOE ε4, apolipoprotein E ε4.

To better understand the group-level differences in FA, MD, AD, RD and microstructural alterations (FW, FAt), we compared former American football players and the unexposed asymptomatic controls using a voxel-wise TBSS analysis.^53^ With a significance threshold of 0.99, or a *P* < 0.01, there was higher FA in the former football players in 1.97% of the skeleton (1552 voxels), spread across the following locations: anterior limb of the internal capsule, external capsule, superior corona radiata and superior frontal-occipital fasciculus (Cohen’s *d* = 0.587) (see Fig. 1). Additionally, we found higher FAt in the former football players in 1.48% of the skeleton (1004 voxels) across the following locations: anterior limb of the internal capsule, external capsule, superior corona radiata, superior frontal-occipital fasciculus and the uncinate fasciculus (Cohen’s *d* = 0.616) (see Figs 1 and 2). No significant differences were found for MD, AD, RD, or FW between groups (all *P*’s > 0.05).

#### Voxel-wise comparisons by traumatic encephalopathy syndrome diagnosis

To better understand the group-level differences in FA, MD, AD, RD and microstructural alterations (FW, FAt), we compared football players with and without a TES diagnosis. There were no group-level differences (all *P*’s > 0.07) in the white matter skeleton between those with and without TES.

### Age and exposure-related associations with diffusion metrics

Using diffusion values extracted from the TBSS white matter skeleton, we examined associations between FA/FAt and exposure-related variables. We found that whole-brain FA (*P* < 0.00001) and FAt (*P* < 0.00001) decreased with age in both former football players and controls. Among football players, FA (*P* < 0.01) and FAt (*P* < 0.01) were significantly lower with earlier age at first exposure to football. Additionally, there was a correlation between FAt in former football players and estimates of head impact exposure, as measured by CHII scores, such that we observed FAt decreases with higher CHII linear acceleration (*P* < 0.04) and higher rotational forces (*P* < 0.02). *Post hoc* analysis examining MD, RD and AD associations with age and exposure factors did not reveal any significant interactions (all *P*’s > 0.9). See Table 2 for a summary of age and exposure-related associations with FA and FAt.

**Table 2:** Linear regression parameter estimates for associations between diffusion metrics and exposure-related variables in former football players

Outcome	Predictor	β (estimate)	95% CI	*P* (uncorrected)	*P* (corrected)
FA	Age	−0.001	[0.001–0.0008]	< 0.00001	<0.00001
FA	Age of first exposure	0.0016	[0.0006–0.0026]	< 0.01	<0.01
FA	CHII-frequency	−0.00000012	[−0.0000007–0.0000005]	0.7	0.7
FA	CHII-linear acceleration	−0.00000002	[−0.00000006–0.00000001]	0.2	0.3
FA	CHII-rotational force	−0.0000000002	[−0.0000000007–0.0000000002]	0.2	0.3
FAt	Age	−0.0006	[0.001–0.0004]	<0.00001	<0.00001
FAt	Age of first exposure	0.001	[0.0005–0.002]	<0.01	<0.01
FAt	CHII-frequency	−0.0000002	[−0.0000006–0.0000002]	0.3	0.3
FAt	CHII-linear acceleration	−0.00000003	[−0.00000006–−0.000000007]	0.01	0.02
FAt	CHII-rotational force	−0.0000000003	[−0.0000000007–−0.00000000004]	0.02	0.04

Unstandardized regression coefficients (β) and 95% confidence intervals are shown for linear models examining associations between whole-brain FA and FAt and exposure-related variables, including age, age of first exposure to tackle football and CHII metrics (frequency, linear acceleration, and rotational force). *P*-values are reported both uncorrected and after Benjamini–Hochberg correction for multiple comparisons. FA and FAt values were averaged across the TBSS white matter skeleton before analysis.

## Discussion

From the TBSS analysis, we found greater whole-brain FA and FAt in the white matter skeleton of our former American football players than in controls. The differences were most prominent in the anterior limb of the internal capsule, external capsule, superior corona radiata, superior frontal-occipital fasciculus and uncinate fasciculus. Whole-brain FA and FAt declined significantly with age in both football players and controls. Among football players, the earlier age of first exposure to football was associated with lower FA and FAt. Furthermore, FAt was negatively associated with estimated head impact exposure, with significant reductions observed in both linear and rotational CHII metrics. There were no significant differences in AD, RD, or MD between groups. In our TES analysis, we found no significant group-level differences in white matter microstructure (FA, MD, AD, RD, FW, FAt) between former football players with and without a TES diagnosis. No significant associations were found between MD, RD, or AD and age or exposure metrics.

These group-level differences, specifically the elevated FA and FAt observed in former football players compared to controls, add to the growing but inconsistent body of literature examining white matter microstructure in athletes exposed to RHI. Although a recent systematic review concluded that RHI is often associated with reduced FA,^38^ prior studies have yielded inconsistent results.^28,54-57^ Some have reported elevated FA in athletes with greater cumulative contact sport exposure or a history of concussion, as seen in cohorts of high school and collegiate athletes,^28^ collegiate athletes with prior concussion,^54^ and professional football players reporting multiple concussions.^55^ Similarly, TBSS studies in hockey players and longitudinal studies in concussed athletes have shown increased FA and reduced RD or MD in select regions.^56^ In contrast, other investigations, including those in retired professional football or hockey players,^57^ have reported no significant microstructural differences relative to controls. Taken together, the literature and our current findings suggest that diffusion changes following RHI may reflect a heterogeneous set of neurobiological responses, potentially influenced by age, exposure timing, cumulative burden and individual variability in resilience or repair mechanisms. We dive further into this below.

While FA is commonly interpreted as a proxy for white matter integrity, it is important to recognize that this metric reflects a complex interplay of microstructural features, including axonal density, myelination and the coherence of fibre orientation.^58-65^ Increases in FA are not always indicative of improved or healthier tissue, just as decreases do not necessarily reflect damage.^66^ FA is particularly sensitive to underlying fibre architecture and cellular organization, and in regions of complex crossing fibres, changes in FA may reflect alterations in fibre geometry rather than degeneration or repair.^66-68^ Importantly, our primary effects were localized to core regions of the TBSS white matter skeleton, which may be less influenced by extreme peripheral crossing-fibre configurations, although tensor-based approaches cannot fully resolve fibre orientation complexity. Thus, the elevated FA observed in former football players in our study may not reflect preserved or enhanced white matter integrity, but rather shifts in underlying microstructure, such as gliosis, selective vulnerability of crossing fibres, or compensatory reorganization, that alter diffusion anisotropy without overt tissue loss. These considerations underscore the importance of interpreting FA within the broader context of complementary diffusion metrics (e.g. RD, AD, MD) and advanced modelling approaches.

Many of the regions where we observed elevated FA and FAt, including the superior corona radiata, superior fronto-occipital fasciculus and anterior limb of the internal capsule, are characterized by complex fibre architecture and are particularly susceptible to crossing fibres.^69,70^ In such regions, elevated FA may reflect selective disruption of crossing tracts or reorganization of dominant pathways, rather than increased white matter integrity. Similarly, regions like the uncinate and external capsule are vulnerable to diffuse axonal injury and may exhibit changes in FA due to gliosis, neuroinflammatory responses, or microstructural compensation. These complexities further highlight the need for caution when interpreting diffusion metrics in isolation.

Regions with higher FA/FAt in former players reflect training-related enhancements in white matter organization rather than preserved tissue in the face of injury.^71-75^ Our control group comprised non-athletes; thus, the between-group contrast may conflate athlete-to-non-athlete differences (for example, long-term sport practice, visuomotor skill acquisition and conditioning) with exposure-related effects. This possibility is particularly salient for tracts subserving visuomotor integration and motor planning, such as the superior fronto-occipital fasciculus, superior corona radiata and anterior limb of the internal capsule, which undergo high, sport-specific loading and intensive skill use.^74^ It may be the case that a positive shift in anisotropy associated with sport training and selection into elite play (yielding higher FA/FAt versus non-athlete controls in some regions), overlaid with a dose-dependent degradation linked to RHI burden (the negative associations of FA/FAt with earlier age of first exposure and cumulative kinematics within players), can explain the pattern of our results.^71-75^ While our primary interpretation emphasizes microstructural compromise with increasing exposure, it is important to acknowledge that increased white matter integrity from athletic training remains a plausible contributor to elevated FA/FAt in select pathways. Future work that includes athlete control groups, objective fitness indices and complementary microstructural measures will help disentangle training-related adaptations from exposure-related injury.

We next examined differences in white matter microstructure between former football players who met diagnostic criteria for TES and those who did not. No significant differences were observed across any diffusion metric—FA, FAt, MD, AD, or RD—between the two groups. These findings suggest that diffusion-based measures of white matter microstructure may not distinguish TES status among individuals with similar exposure to RHI. Importantly, former football players with and without a TES diagnosis in our sample had histories of substantial RHI exposure, which may contribute to a shared neurobiological profile that is not fully captured by symptom-based classification alone. Our results underscore the complexity of linking clinical symptomatology with imaging biomarkers and highlight the need for continued research that integrates clinical, biological and exposure-based data. As efforts to validate and refine the TES criteria evolve, incorporating objective markers of neurodegeneration may enhance its utility in identifying individuals at elevated risk for long-term neurological consequences following RHI.

Our findings should also be considered alongside recent DIAGNOSE CTE Research Project work examining diffusion measures at the grey matter/white matter boundary.^76^ That study emphasized microstructural properties immediately beneath the cortex, motivated by the preferential localization of CTE p-tau pathology near sulcal depths, and reported associations between higher boundary FA, TES-related features and CSF p-tau measures. In contrast, the current analyses focus on central white matter microstructure and show that any group-level differences are modest and spatially restricted and that TES-related differences are not robust within the TBSS skeleton. Together, these findings suggest that diffusion effects relevant to TES/CTE may be more detectable near the GM/WM boundary than in central white matter, underscoring the importance of neuroanatomically targeted diffusion approaches.

Given the variability in findings on white matter microstructure following RHI, we examined specific exposure-related factors beyond general participation in contact sports. Among former football players, we assessed associations between diffusion metrics and chronological age, age of first exposure to tackle football and cumulative head impact exposure estimated using CHII metrics. As expected, both FA and FAt declined with age, consistent with known effects of normal ageing.^77^ While we did not evaluate formal age-by-exposure interactions, these results highlight the importance of accounting for age-related variability when interpreting exposure-associated diffusion differences in former athletes. We also found that players with an earlier age of first exposure had lower FA and FAt later in life, even after accounting for total years of play. This aligns with previous studies showing that earlier age of first exposure is associated with poorer cognitive outcomes, reduced cortical thickness and greater symptom severity in older age.^8,22-24,78-80^ Because the present cohort consists of elite former football players with high cumulative exposure, it will be important for future work to determine whether similar age-of-first-exposure associations are observed in athletes with lower levels of contact sport participation. In addition, greater estimated linear and rotational head impacts were linked to lower FAt, suggesting that more forceful impacts contribute to lasting microstructural alterations. These findings emphasize that both the timing and intensity of RHI exposure may influence long-term white matter health and support the use of CHII metrics in modelling individual risk. They support the importance of implementing age-appropriate safety measures in youth football, while acknowledging that not all individuals exposed to RHI will experience adverse outcomes.

### Limitations

While this study offers important insights into the effects of RHI on white matter microstructure, several limitations should be noted. Our control group comprised non-athletes, which may introduce variability related to differences in physical activity, cardiovascular health and lifestyle factors. Future studies incorporating athletic controls would help better isolate the specific impact of head trauma. Additionally, although diffusion metrics such as FA and FAt provide valuable information, they are influenced by factors like crossing fibres and complex tissue architecture, which can complicate interpretation. The TBSS method, while robust for group comparisons, focuses on the core of major white matter tracts and may not capture more subtle or peripheral microstructural changes. We also note a lateralization within our results; however, we do not have sufficient data to investigate this further (e.g. handedness, head injury locations), so therefore, any assumption we make will be speculative. Our sample was also limited to male participants, restricting generalizability to female athletes. Despite these limitations, the current findings highlight meaningful associations between specific exposure-related factors and white matter alterations.

## Conclusion

Our findings contribute to the growing and complex literature on the effects of RHI on white matter microstructure in former athletes who participated in contact sports. We observed elevated FA and FAt in several major white matter tracts among former football players compared to controls. White matter alterations were associated with specific exposure-related factors, including increasing age, earlier age of first exposure to tackle football and greater estimated cumulative head impact burden. Our measures did not differentiate individuals with or without a TES diagnosis.

## Supplementary Material

fcag195_Supplementary_Data

## Data Availability

Data from the DIAGNOSE CTE Research Project will be shared with qualified investigators via the Federal Interagency Traumatic Brain Injury Research (FITBIR) Informatics System hosted by the NIH Center for Information Technology (see access instructions: https://fitbir.nih.gov/content/access-data). The materials used in this study were derived from controlled-access resources supported by the US Department of Defense (DoD) and the National Institutes of Health (NIH); this national informatics infrastructure was created to accelerate research in traumatic brain injury and related trauma. The opinions expressed here are those of the authors and do not necessarily reflect the views of the DoD, NIH, or the original data contributors. Inquiries about the data presented in this manuscript may be directed to H.A. via email at Hector.Arciniega@nyulangone.org.

## Appendix

**DIAGNOSE CTE Research Project Team**

Eric Reiman, M.D.

Yi Su, Ph.D.

Kewei Chen, Ph.D.

Hillary Protas, Ph.D.

Connie Boker, M.B.A.

Michael L. Alosco, Ph.D.

Rhoda Au, Ph.D.

Robert C. Cantu, Ph.D.

Lindsay Farrer, Ph.D.

Robert Helm, M.D.

Douglas I. Katz, M.D.

Neil Kowall, M.D.

Jesse Mez, M.D.

Gustavo Mercier, M.D., Ph.D.

James Otis, M.D.

Robert A. Stern, Ph.D.

Jason Weller, M.D.

Irene Simkin, M.S.

Alondra Andino, B.A.

Shannon Conneely, B.A.

Courtney Diamond, M.B.A.

Tessa Fagle, B.A.

Olivia Haller, B.A.

Tennyson Hunt, M.B.A.

Nicole Gullotti, M.B.A.

Megan Mariani, B.S., B.A.

Brian Mayville, B.S.

Kathleen McLaughlin, B.A.

Mary Nanna, B.A.

Taylor Platt, M.P.H.

Surya Pulukuri, B.A.

Fiona Rice, M.P.H.

Madison Sestak, B.S.

Michael McClean, Sc.D.

Yorghos Tripodis, Ph.D.

Douglas Annis, M.S.

Christine Chaisson, M.P.H.

Diane B. Dixon

Carolyn Finney, B.A.

Kerrin Gallagher, M.P.H.

Kaitlin Hartlage, M.P.H.

Jun Lu, M.S.

Brett Martin, M.S.

Emmanuel Ojo, M.P.H.

Joseph N. Palmisano, M.A., M.P.H.

Brittany Pine, B.A., B.S.

Janani Ramachandran, M.S.

Sylvain Bouix, Ph.D.

Jennifer Fitzsimmons, M.D.

Alexander P. Lin, Ph.D.

Inga K. Koerte, M.D., Ph.D.

Ofer Pasternak, Ph.D.

Martha E. Shenton, Ph.D.

Hector Arcinieago, Ph.D.

Tashrif Billah, M.S.

Elena Bonke, M.S.

Katherine Breedlove, Ph.D.

Eduardo Coello, Ph.D.

Michael J. Coleman, M.A.

Leonhard Jung

Huijun Liao, B.S.

Maria Loy, M.B.A., M.P.H.

Elizabeth Rizzoni, B.A.

Vivian Schultz, M.D.

Annelise Silva, B.S.

Brynn Vessey, B.S.

Tim L.T. Wiegand

Sarah Banks, Ph.D.

Charles Bernick, M.D.

Jason Miller, Ph.D.

Aaron Ritter, M.D.

Marwan Sabbagh, M.D.

Raelynn de la Cruz

Jan Durant

Morgan Golceker

Nicolette Harmon

Kaeson Kaylegian

Rachelle Long

Christin Nance

Priscilla Sandoval

Robert W. Turner, Ph.D.

Kenneth L. Marek, M.D.

Andrew Serrano, M.B.A.

Charles H. Adler, M.D., Ph.D.

David W. Dodick, M.D.

Yonas Geda, M.D., MSc

Jennifer V. Wethe, Ph.D.

Bryce Falk, R.N.

Amy Duffy

Marci Howard

Michelle Montague

Thomas Osgood

Debra Babcock, M.D., Ph.D.

Patrick Bellgowan, Ph.D.

Laura Balcer, M.D., M.S.C.E.

William Barr, Ph.D.

Judith Goldberg, Sc.D.

Thomas Wisniewski, M.D.

Ivan Kirov, Ph.D.

Yvonne Lui, M.D.

Charles Marmar, M.D.

Lisena Hasanaj

Liliana Serrano

Alhassan Al-Kharafi

Allan George

Sammie Martin

Edward Riley

William Runge

Jeffrey L. Cummings, M.D., ScD

Elaine R. Peskind, M.D.

Elizabeth Colasurdo

Daniel S. Marcus, Ph.D.

Jenny Gurney, M.S.

Richard Greenwald, Ph.D.

Keith A. Johnson, M.D.
