# Supplementary material for: Dose-dependent white matter changes associated with repetitive head impacts in former American football players
Source: Brain Commun. 2026 May 31;8(3):fcag195. doi: 10.1093/braincomms/fcag195 (PMC13253572; doi:10.1093/braincomms/fcag195)
Supplement: fcag195_Supplementary_Data [file fcag195_supplementary_data.pdf]

**Inclusion**	**Exclusion**
**Overall Criteria**	
- Male, age 45-74<br>- No contraindications to procedures performed in the DIAGNOSE CTE Research Project<br>- English as primary language<br>- Willingness and ability to consent to study participation of the participants and a study partner	- History of clinical Stroke or significant neurological condition, severe enough to compromise neuropsychological testing<br>- Other clinically significant organ disease (e.g. liver, kidney...)
**Former professional American football players**	
- ≥12 years of organized football (≥3 in college; ≥4 in the NFL)	- Participants were excluded if their football participation was limited to quarterback, kicker, punter or special teams only
**Former collegiate American football players**	
- ≥6 years of organized football (≥3 in college)	- Participants were excluded if their football participation was limited to quarterback, kicker, punter or special teams only
**Unexposed Participants**	
- No history of participation in organized contact/collision sport or military service and training<br>- Asymptomatic based on telephone screening regarding neuropsychological and psychiatric conditions<br>- At least two years of secondary education	- History of TBI, Concussion<br>- History of formal diagnosis of psychiatric or neurological illness

Advertisement

All Participants: Newspaper advertisement, billboard advertisement and social media posts.
Football targeted advertisement via NFL alumni groups and videos of Super Bowl champion Ben Utrecht.

↓

DIAGNOSE CTE Research Project Sample
(Cross-Sectional)

N = 180 Former American football players
- N = 120 Former professional football players
- N = 60 Former collegiate football players

N = 60 Unexposed Participants

↓

Exclusion due to data quality control

N = 15 Former American football players
- N = 15 (Missing or poor quality T1w or T2w MRI)

N= 8 Unexposed Participants
- N = 4 (Missing or poor quality T1w or T2w MRI)
- N = 2 (Undisclosed RHI exposure)
- N = 2 (Undisclosed excluding illness)

↓

Study Sample
(Cross-Sectional)

N = 165 Former American football players
- N = 111 Former professional football players
- N = 54 Former collegiate football players

N = 52 Unexposed Participants

Supplementary Figure 1. Inclusion and exclusion criteria of the DIAGNOSE CTE research project and the data included in this study.

Abbreviations: NFL, National Football League; TBI, traumatic brain injury; MRI, magnetic resonance imaging.
